# MicroRNA-497-5p Is Downregulated in Hepatocellular Carcinoma and Associated with Tumorigenesis and Poor Prognosis in Patients

**DOI:** 10.1155/2021/6670390

**Published:** 2021-03-16

**Authors:** Lin-Lin Tian, Bin Qian, Xiao-Hui Jiang, Yu-Shan Liu, Tong Chen, Cheng-You Jia, Ya-Li Zhou, Ji-Bin Liu, Yu-Shui Ma, Da Fu, Sen-Tai Ding

**Affiliations:** ^1^Department of Urology, Shandong Provincial Hospital Affiliated to Shandong First Medical University, Jinan 250021, China; ^2^Department of Microbiology, Faculty of Basic Medical Sciences, Guilin Medical University, Guilin 541001, China; ^3^Department of General Surgery, Shanghai Eighth People's Hospital, Shanghai 200235, China; ^4^Department of Gastrointestinal Surgery, Affiliated Tumor Hospital of Nantong University, Nantong 226631, China; ^5^Department of Pathology, Nantong Tumor Hospital, Nantong 226631, China; ^6^Department of Pediatric Surgery, Shanghai Children's Hospital, Shanghai Jiao Tong University, China; ^7^Central Laboratory for Medical Research, Shanghai Tenth People's Hospital, Tongji University School of Medicine, Shanghai 200072, China

## Abstract

**Background:**

MicroRNAs (miRNAs) have been demonstrated to exhibit important regulatory roles in multiple malignancies, including hepatocellular carcinoma (HCC). hsa-miR-497-5p was reported to involve in cancer progression and poor prognosis in many kinds of tumors. However, the expression and its clinical significance of hsa-miR-497-5p in HCC remain unclear.

**Methods:**

In the present study, we investigated the expression of hsa-miR-497-5p in HCC and analyzed the correction of clinical features with prognosis. The expression levels of hsa-miR-497-5p and potential target genes were analyzed in HCC and adjacent noncancerous tissues using The Cancer Genome Atlas (TCGA) database and Gene Expression Omnibus (GEO) datasets. Real-time quantitative reverse transcription polymerase chain reaction (qRT-PCR) was used to analyze hsa-miR-497-5p levels in 328 HCC tissues and 30 paired adjacent noncancer tissues. Overall survival (OS) and progression-free survival (PFS) of patients with HCC were assessed using the Kaplan-Meier method and the log-rank test.

**Results:**

The hsa-miR-497-5p expression levels were decreased, and its target genes ACTG1, CSNK1D, PPP1CC, and BIRC5 were upregulated in HCC tissues compared with normal tissues. Lower levels of hsa-miR-497-5p expression and higher levels of the four target genes were significantly associated with higher tumor diameter. Moreover, patients with lower hsa-miR-497-5p expression and higher target genes levels had shorter OS.

**Conclusion:**

The expression levels of hsa-miR-497-5p may play an important regulatory role in HCC and are closely correlated with HCC progression and poor prognosis in patients. The hsa-miR-497-5p may be a specific therapeutic target for the treatment of HCC.

## 1. Introduction

Hepatocellular carcinoma (HCC), representing approximately 90% of total primary liver cancer, is the second most frequent cause of cancer-associated deaths worldwide [1–3]. Several factors play essential roles during the progression of HCC, including the tumor microenvironment and genetic mutations [4–6]. Despite various treatment options, HCC is of high incidence and mortality due to rapid proliferation, cancer recurrence, and distant metastasis [7–10]. Thus, identification of novel treatment targets or useful biomarkers is badly needed to improve the clinical treatment outcomes of HCC.

MicroRNAs (miRNAs), a widespread small noncoding endogenous RNA, can interact with target messenger RNA (mRNA) to regulate target gene expression at the posttranscriptional level, influencing many important biological processes such as cell proliferation, invasion, and tumorigenesis [11–13]. Plenty of researches have shown that miRNA was closely intertwined with HCC occurrence and progression [14–16]. Furthermore, some studies suggest that miRNA play roles as either oncogenes or tumor suppressors, which can be used as predictive biomarkers or therapeutic targets in clinical applications [17–20].

hsa-miR-497-5p is located in human chromosome 17 (17p13.1) and derived from the miR-15 family [21]. hsa-miR-497-5p is widely distributed in the body, and its expression level is strongly associated with a poor prognosis and tumor progression [22–24]. It is shown that overexpression of hsa-miR-497-5p can regulate the expression of Smad3 and lead to cell cycle arrest in the G0/G1 phase [25–27]. Overexpression of miR-497-5p can reduce the content of insulin receptors and induce insulin resistance in HFD-MES rats [28–30]. Furthermore, the expression of miR-497-5p is associated with the occurrence and development of HCC. Zhang et al. reported that miR-497-5p was upregulated in HCC samples, and the high expression of miR-497-5p leads to the increase of tumor size and number. What is more, silencing of miR-497-5p could suppress HCC cell proliferation and migration [31]. On the contrary, Xu et al. reported that miR-497-5p was downregulated in HCC clinical samples and cell lines, and low expression of miR-497-5p was associated with aggressive tumor characteristics as well as patients' prognosis in HCC patients [32]. Therefore, it is still controversial whether miR-497-5p is an oncogene or a tumor suppressor gene in HCC.

Here, we found that downregulation of miR-497-5p is closely associated with tumorigenesis and poor prognosis in HCC. The study is aimed at analyzing the expression of miR-497-5p and its potential target genes as well as its efficacy on overall survival (OS) and progression-free survival (PFS) in patients with HCC by bioinformatics analysis and reverse transcription quantitative polymerase chain reaction (RT-qPCR) to discuss its potential clinical significance in this disease.

## 2. Materials and Methods

### 2.1. Patients and Samples

Fresh HCC tumor tissue samples and adjacent normal tissues were collected via surgical resection between August 2004 and September 2014. A total of 328 primary HCC samples were collected, including tumor and adjacent nontumor liver tissues, 30 of which were paired with adjacent normal tissues. This study has been approved by the institutional research ethics committee of Shanghai Tenth People's Hospital (Approval No. SHSY-IEC-2019 K10). All the participants provided written informed consent prior to investigation in the study.

### 2.2. Data Retrieval and Download

RNA expression data of HCC patients in TCGA (https://cancergenome.nih.gov/) and GEO (https://www.ncbi.nlm.nih.gov/gds/) databases were chosen for analysis. The sequencing data of miRNA expression of 372 HCC tissues and 50 normal tissues were procured from TCGA. miRNA data from three original GEO datasets (GSE36915, GSE97098, and GSE31383) were downloaded. The expression of hsa-miR-497-5p in HCC tissues and adjacent normal tissues was analyzed. The Cancer Cell Line Encyclopedia (CCLE, https://portals. http://broadinstitute.org/ccle) was used to verify the expression and DNA methylation of miR-497-5p in tumor cell lines.

### 2.3. DEGs Screening and Survival Analysis

The DEGs between HCC samples and noncancerous tissues were determined using the EdgeR software package [33]. Fold change (FC) ≥ 2 or ≤0.5 and *p* < 1.0*E* − 10 were chosen as elementary screening arguments for cluster analysis. Hierarchical clustering was used to screen DEGs by the multiple experiment viewer (MeV) 4.7.1 software program (http://www.tm4.org). After that, we conducted a survival analysis, and the analysis was performed using GraphPad Prism 8.0.1 (http://www.graphpad.com/) software.

### 2.4. RNA Isolation and Quantitative Reverse Transcriptase PCR

Total RNA was isolated from HCC cell lines, tumor samples, and normal tissue samples by the TRIzol reagent (Invitrogen, Waltham, MA, USA), according to the manufacturer's guide. Obtaining cDNA from total RNA by reverse transcription and qRT-PCR of miR-497-5p was obtained using the TaqMan miRNA qRT-PCR Kit (HaiGene, Haerbin, China). Each sample shall be tested independently in at least triplicate. U6 small nuclear RNA was used for an internal control. The expression level of miR-497-5p and four potential target genes in each sample was calculated and expressed as *^Δ^*Ct value, and the 2^−*ΔΔ*CT^ method was applied to analyze the relative hsa-miR-497-5p expression levels.

### 2.5. Cell Lines

Human liver cancer lines Huh7, Hep3B, and HepG2 were purchased from the Cell Bank of the Chinese Academy of Sciences (Shanghai, China) and cultured in DMEM media (Invitrogen, Carlsbad, USA) and supplemented with 10% (*v*/*v*) fetal bovine serum (FBS), 100 U/ml penicillin, and 100 mg/ml streptomycin. Cell lines were routinely tested for mycoplasma contamination and have been authenticated with short-tandem repeat analysis. Cell culture was conducted at 37°C in a humidified 5% CO_2_ incubator.

### 2.6. Cell Proliferation Assays

For the cell proliferation assays, 1,000 cells were seeded into 96-well plates to culture overnight. Next, the CCK8 (10 l) reagent was then added to each well, the absorbance (*A*) was measured at 450 nm after 1 h, and the relative cell viability rate was calculated. All experiments were performed in triplicate.

### 2.7. Bioinformatics Analysis

Hierarchical clustering was performed using the multiple experiment viewer (MeV) 4.7.1 software programs (http://www.tm4.org/mev/). We used online target gene prediction software DIANA-miRPath (http://http://snf-515788.vm.okeanos.grnet.gr/), miRDB (http://mirdb.org/miRDB/), miRTarBase (http://mirtarbase.mbc.nctu.edu.tw/), and miRanda (http://www.microrna.org) to forecast several potential target genes of hsa-miR-497-5p. Subsequently, predicted target genes were subjected to KEGG pathway online analysis. The expression levels of four potential target genes in LIHC were obtained from GEPIA (http://gepia.cancer-pku.cn/).

### 2.8. Statistical Analysis

Statistical analysis was performed by GraphPad Prism (http://www.graphpad.com/) Software. Overall survival (OS) and progression-free survival (PFS) were evaluated by the Kaplan-Meier curves, and differences between survival rates were examined using the log-rank test. OS was calculated from the first day of diagnosis until the time of last follow-up or death. PFS was assessed as the interval from random assignment to the first documented disease progression or death because of any cause.

## 3. Results

### 3.1. Cluster Analysis of miRNA Expression in HCC

**Figure 1:**
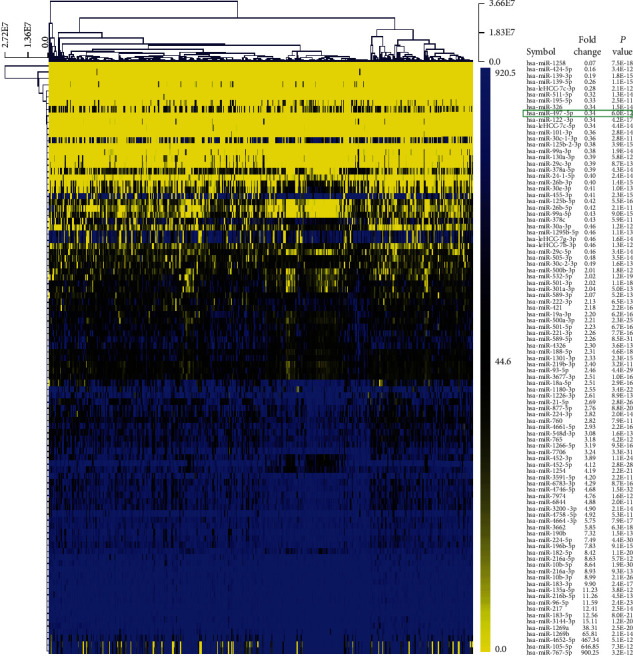
Cluster analysis of miRNA expression in HCC. Cluster analysis of miRNA expression in HCC was performed by MeV software (version 4.7.1).

A total of 2,166 miRNA-seq data of 372 patients with LIHC cancer tissues (cancer) and 50 cases of adjacent normal tissue (normal) were downloaded from TCGA. The genes meet the criteria of fold change (FC) ≥ 2 or ≤0.5 and *p* value < 1.0*E*-10. 95 DEGs were selected between LIHC and control samples, which were composed of 62 upregulated genes and 33 downregulated genes. The heat map for hierarchical clustering analysis was acquired by using MEV4.7.1 software (Figure 1).

### 3.2. The Expression and Prognosis of DEGs in HCC

**Figure 2:**
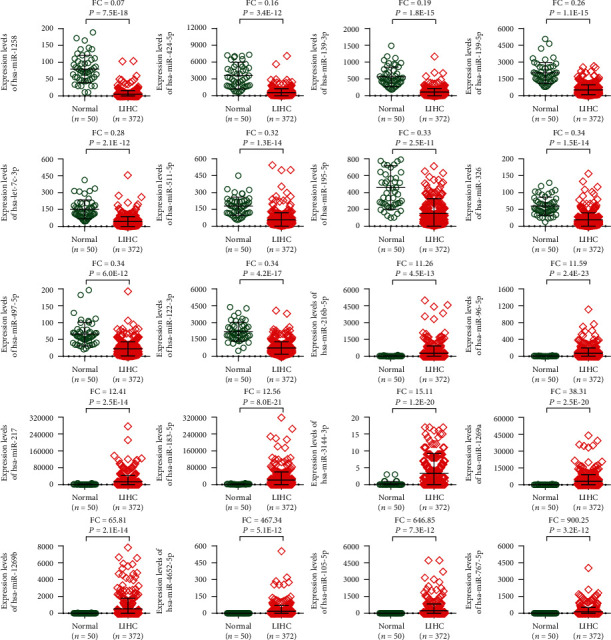
The expression level of top 20 DEGs in HCC. The genes meet the criteria of fold change (FC) ≥ 2 or ≤0.5 with *p* value < 1.0*E*-10.

According to fold change, as indicated in Figure 2, the expression level of miRNA in the top 20 DEGs (top 10 up- and downregulated genes) of LIHC tissues was compared with their corresponding nontumor normal tissues.

**Figure 3:**
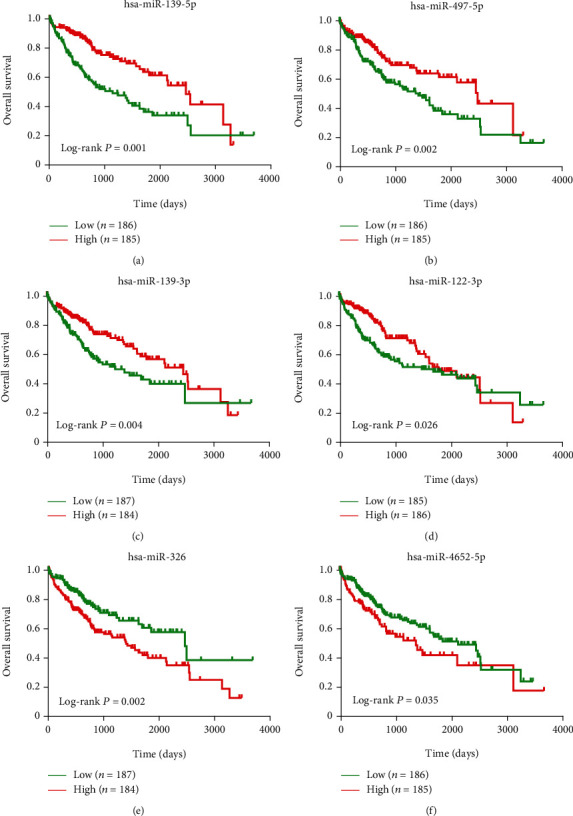
Six of the top 20 DEGs associated with the prognosis of HCC patients. Overall survival (OS) of the top 20 DEGs was evaluated by the Kaplan-Meier curves, and differences between survival rates were examined using the log-rank test.

In addition, there was a total of 372 LIHC samples with complete overall survival (OS) information; six of the top 20 DEGs are associated with the prognosis of patients (Figure 3).

**Figure 4:**
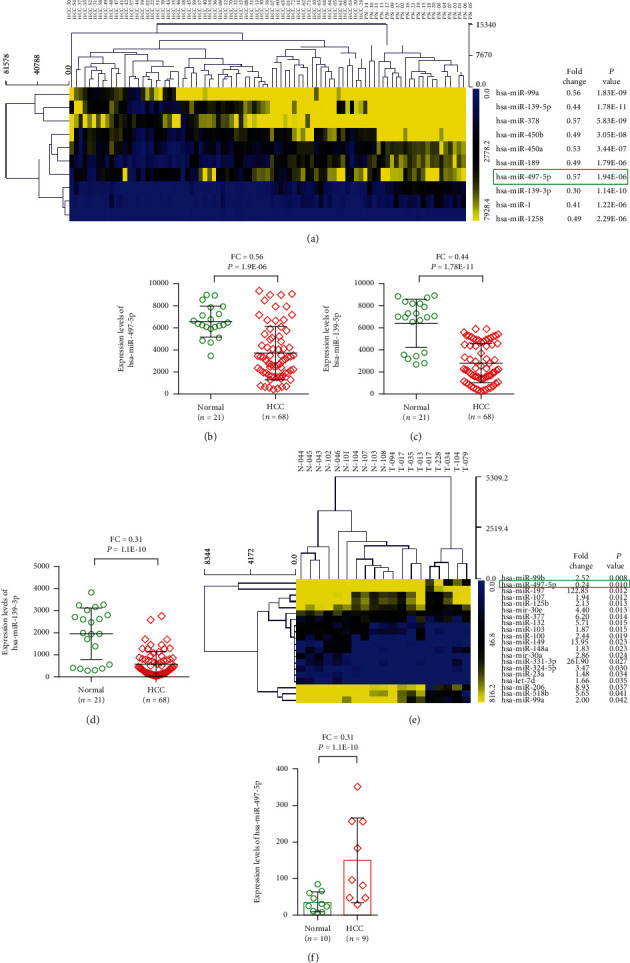
The levels of miR-497-5p expression in HCC samples. (a) The top ten DEGs miRNAs were screened out using MEV4.7.1 software. miR-497-5p (b), miR-139-5p (c), and miR-139-3p (d) were also highly expressed in tumor tissues compared with normal tissue. (e) The top twenty DEGs were selected between HCC and control samples. (f) The expression level of miR-497-5p was significantly higher in HCC tissue compared with nontumor tissue.

Next, the raw data were extracted from the GEO database (GEO accession no. GSE36915). In this present study, we examined the expression levels of miRNAs in tumor tissues and normal tissue from a group of 89 patients with HCC. The top ten DEGs miRNAs were screened out using MEV4.7.1 software (Figure 4(a)). As a result, we found that three DEGs (hsa-miR-497-5p, hsa-miR-139-5p, and hsa-miR-139-3p) were also as significant DEGs in the TCGA-LIHC database. Moreover, the three DEGs were also highly expressed in tumor tissues compared with normal tissue (Figures 4(b)–4(d)).

To further identify the dependability of the miRNA genes, the same analysis was used to verify the next dataset from the NCBI GEO database (GEO accession no. GSE31383). The top twenty DEGs were selected between HCC and control samples (Figure 4(e)). Our results demonstrated that the expression level of miR-497-5p was significantly higher (*p* = 1.1*E* − 10) in HCC tissues compared with nontumor tissues (Figure 4(f)).

### 3.3. The Levels of miR-497-5p Expression in HCC Samples

**Figure 5:**
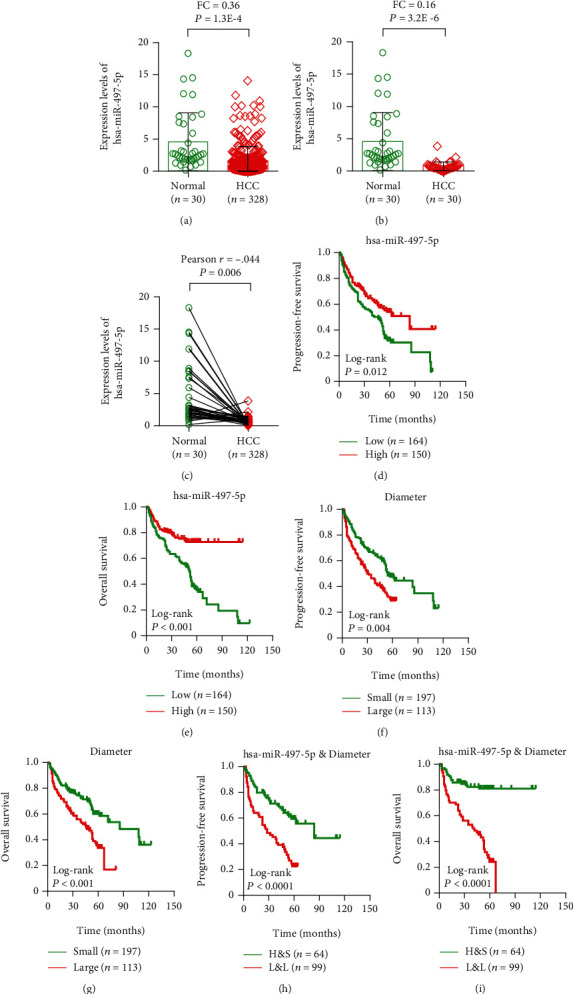
Association between miR-497-5p expression and clinical features with HCC prognosis. (a) The expression levels of miR-497-5p were significantly downregulated contrasted with adjacent noncancerous tissues. (b) A significantly lower miR-497-5p in human HCC specimens compared with adjacent noncancerous specimens. (c) Pearson correlation miR-497-5p in HCC tumor tissues in contrast with adjacent normal tissues. (d, e) Lower miR-497-5p expression was significantly correlated with poor PFS (d) and OS (e). (f, g) Tumor diameter was significantly correlated with decreased duration of PFS (f) and OS (g) of patients with HCC. (h, i) Patients with low miR-497-5p levels and large tumor diameter (≥5 cm) were associated with shorter PFS (h) and OS (i).

The levels of miR-497-5p expression were evaluated in HCC samples (*n* = 328) contrasted with normal samples (*n* = 30) by qRT-PCR. In HCC tissue samples, the results indicated that the expression levels of miR-497-5p were significantly downregulated contrasted with adjacent noncancerous tissues (*p* = 1.3*E* − 4; FC, 0.36; Figure 5(a)).

To evaluate the miR-497-5p expression levels divided between HCC and normal tissues, we detected the expression in 30 pairs of tumor tissues and adjacent nonneoplastic liver tissues using qRT-PCR. In particular, the present results indicated a significantly lower miR-497-5p in human HCC specimens compared with adjacent noncancerous specimens (*p* = 3.2*E* − 6; FC, 0.16; Figure 5(b)).

Subsequently, further analysis of the Pearson correlation between miR-497-5p in HCC tumor tissues in contrast with adjacent normal tissues was negatively correlated (*r* = −0.44; Figure 5(c)).

### 3.4. Association between miR-497-5p Expression and Clinical Features with HCC Prognosis

To further identify the prognostic significance of miR-497-5p expression in HCC, GraphPad software was used to assess the association of miR-497-5p expression between progress-free survival (PFS) and overall survival (OS). The results show that lower miR-497-5p expression was significantly correlated with poor PFS (Figure 5(d)) and OS (Figure 5(e)). Hence, miR-497-5p levels were dramatically related to an increased probability of PFS and OS in patients with HCC.

Our results further indicated that tumor diameter was significantly correlated with decreased duration of PFS (Figure 5(f)) and OS (Figure 5(g)) of patients with HCC. Survival analysis revealed that miR-497-5p levels and tumor diameter were also found to be concerned with PFS and OS. It was observed that patients with low miR-497-5p levels and large tumor diameter (≥5 cm) were associated with shorter PFS and OS than those with high miR-497-5p expression and small tumor diameter (Figures 5(h) and 5(i)).

### 3.5. Expression and Biological Role of miR-497-5p in HCC *In Vitro*

**Figure 6:**
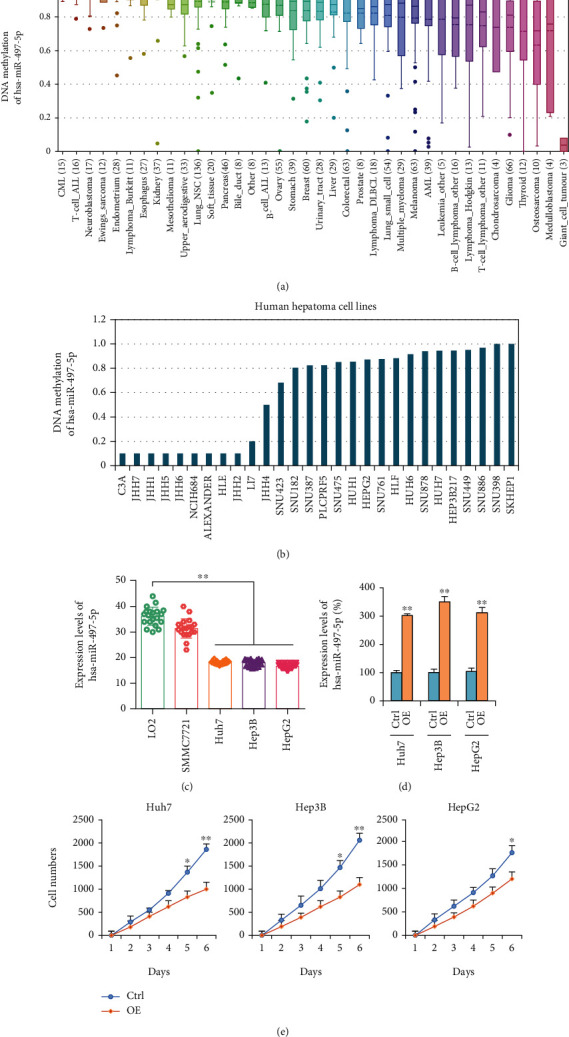
Expression and biological role of miR-497-5p in HCC *in vitro*. (a) DNA methylation of miR-497-5p in cancer cell lines from the Cancer Cell Line Encyclopedia (CCLE) dataset. (b) DNA methylation of miR-497-5p in 28 liver cancer cell lines from CCLE dataset. (c) Expression levels of miR-497-5p in normal liver cell line LO2 and 4 liver cancer cell lines from GEO dataset GSE71108. (d) qRT-PCR measurement of the levels of miR-497-5p mRNA in Huh7, Hep3B, and HepG2 cells before and after overexpression of miR-497-5p. Data shown are the means ± SD of three independent experiments. (e) Huh7, Hep3B, and HepG2 cell counts in 96-well plate after transfection with negative control or miR-497-5p overexpression plasmids at the indicated day. Statistical analyses were performed with one-way ANOVA (^∗^*p* < 0.05, ^∗∗^*p* < 0.01, and ^∗∗∗^*p* < 0.001 vs. normal).

To explore the expression and biological role of miR-497-5p expression in HCC *in vitro*, we performed DNA methylation analysis of miR-497-5p in cancer cell lines from the Cancer Cell Line Encyclopedia (CCLE) dataset and found that DNA methylation analysis of miR-497-5p was low in most tumor cell lines (Figure 6(a)) and liver cancer cell lines (Figure 6(b)). Expression levels of miR-497-5p in liver cancer cell lines Huh7, Hep3B, and HepG2 were significantly lower than that in normal liver cell line LO2 from GEO dataset GSE71108 (Figure 6(c)).

To investigate the biological role of miR-497-5p in HCC cells, we performed gain-of-function studies using miR-497-5p overexpressed plasmid in Huh7, Hep3B, and HepG2 cells (Figure 6(d)). As shown in Figure 6(e), overexpression of miR-497-5p significantly inhibited the growth rate of Huh7, Hep3B, and HepG2 cells transfected with the miR-497-5p overexpressed plasmid compared with the negative control-transfected cells.

### 3.6. Association between miR-497-5p Target Gene Expression and Their Clinical Features with HCC Prognosis

Next, we searched for potential target genes of miR-497-5p using four publicly available target gene prediction databases including DIANA-miRPath, miRDB, miRTarBase, and miRanda to predict the potential target genes of miR-497-5p.

**Figure 7:**
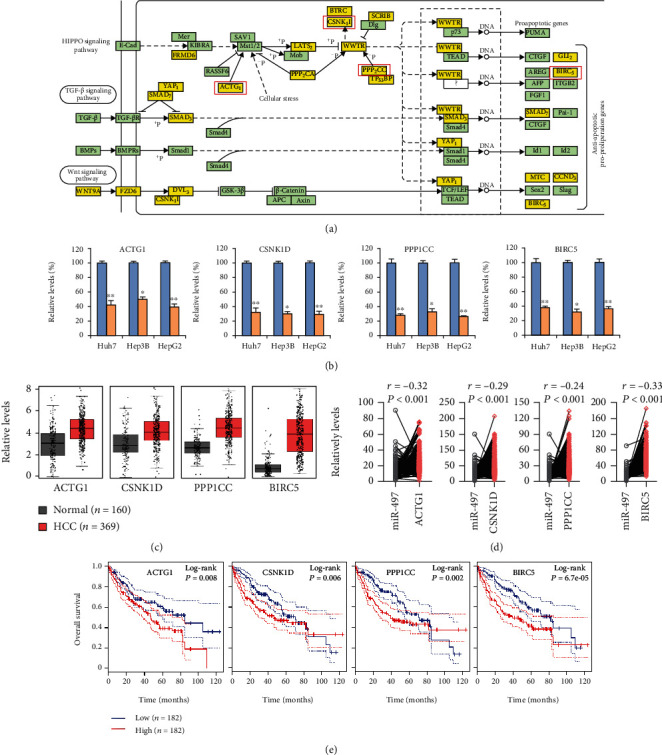
Association between miR-497-5p target gene expression and their clinical features with HCC prognosis. (a) miR-497-5p target prediction using online target gene prediction programs DIANA-miRPath, miRDB, miRTarBase, and miRanda for KEGG analysis of common predicted target genes. (b) qRT-PCR was used to measure the mRNA level of four predicted target genes ACTG1, CSNK1D, PPP1CC, and BIRC5 in Huh7, Hep3B, and HepG2 cells after transfection with negative control or miR-497-5p overexpression plasmids. (c) Expression levels of ACTG1, CSNK1D, PPP1CC, and BIRC5 in normal liver (*n* = 160) and liver cancer (*n* = 369) samples from the GEPIA dataset. (d) Correlation of miR-497-5p expression levels with target genes ACTG1, CSNK1D, PPP1CC, and BIRC5 in liver cancer samples (*n* = 369) from the TCGA dataset. (e) Kaplan-Meier survival analysis to evaluate the prognostic value of ACTG1, CSNK1D, PPP1CC, and BIRC5 expression for OS of HCC patients from the TCGA dataset. Statistical analyses were performed with one-way ANOVA (^∗^*p* < 0.05 and ^∗∗^*p* < 0.01).

The result showed that there were 125 commonly predicted target genes in at least 3 prediction results. Subsequently, predicted target genes were subjected to KEGG pathway analysis, and the results revealed some important cancer and stem-related pathways including the HIPPO signaling pathway, TGFb signaling pathway, and Wnt signaling pathway (Figure 7(a)), in which 4 genes (ACTG1, CSNK1D, PPP1CC, and BIRC5) were clustered in the HIPPO signaling pathway. Moreover, the mRNA expression levels of ACTG1, CSNK1D, PPP1CC, and BIRC5 were significantly lower in the miR-497-5p overexpression group when compared to the control group (Figure 7(b)), which suggested that ACTG1, CSNK1D, PPP1CC, and BIRC5 were regulated by miR-497-5p and were negatively correlated with miR-497-5p.

Next, we analyzed the expression levels of ACTG1, CSNK1D, PPP1CC, and BIRC5 in HCC tissue samples in normal liver (*n* = 160) and liver cancer (*n* = 369) samples from the GEPIA dataset. ACTG1, CSNK1D, PPP1CC, and BIRC5 levels were upregulated in HCC tissues (*p* < 0.01) (Figure 7(c)) when compared with normal liver tissue. Moreover, our results showed that ACTG1, CSNK1D, PPP1CC, and BIRC5 expression was negatively correlated with miR-497-5p expression in HCC tumor biopsies (*n* = 364, *r* = −0.32, -0.29, -0.24, and -0.33, respectively, *p* < 0.001; Figure 7(d)).

We then evaluated the prognostic value of ACTG1, CSNK1D, PPP1CC, and BIRC5 expression for patients with HCC. The OS in HCC patients with a low expression of ACTG1, CSNK1D, PPP1CC, or BIRC5 was prolonged relative to patients with high expression of ACTG1, CSNK1D, PPP1CC, or BIRC5 (Figure 7(e)). Our results indicated that ACTG1, CSNK1D, PPP1CC, and BIRC5 expression was positively correlated with lower OS in HCC patients.

## 4. Discussion

HCC is the fifth most common form of cancer type in the world [33–36]. The traditional treatment strategy for HCC is radical resection, but the rate of recurrence is very high, and the 5-year survival rate is low [37–41]. Thus, the prognosis of HCC is still unsatisfactory. Over the past years, due to the combination of the application of molecular biology techniques and tumor histological examination, great progress has been made in the disease [42–46]. miRNAs are considered an ideal biomarker compared with other biomarkers, miRNAs with sufficient sensitivity in clinical diagnosis and treatment [47–50].

Up to now, many studies have demonstrated that miRNA expression profiles in HCC and nontumor tissues are significantly different [51–54]. Certain miRNAs such as miR-21, miR-221, and miR-222 are frequently overexpressed in HCC [55–62]. These miRNAs have been demonstrated to promote oncogenesis by negatively regulating important tumor-suppressive protein-coding genes in HCC [63–67]. On the other hand, miRNAs such as miR-122, miR-125b, miR-139, miR-101, and let-7 are recurrently downregulated in HCC [68–72]. These miRNAs exert tumor-suppressive effects by repressing oncogenes in HCC [73–75]. miRNA deregulation is an early event that can be detected in premalignant liver dysplastic nodules and can further accumulate during liver carcinogenesis [76–79].

Interestingly, additional studies showed the existence of a large number of stable miRNAs in human serum/plasma, which laid the foundation for studying the role of serum/plasma microRNAs in the diagnosis and prognosis of HCC [80–83]. Differential expression of several serum microRNAs, including miR-16, miR122, miR-21, miR-223, miR-25, miR-375, and let-7f, in patients with HCC, patients with hepatitis B, and healthy individuals was reported recently [84–87].

The aberrant expression of miR-497-5p plays an essential role in tumorigenesis. miR-497-5p has been proven to be downregulated in NSCLC, gastric cancer, colorectal cancer, ovarian cancer, renal cell carcinoma, and melanoma [88–95]. Furthermore, in vitro and in vivo studies revealed that miR-497-5p overexpression suppressed HCC cell proliferation, colony formation, and metastasis [96]. Thus, for a more comprehensive understanding of the clinical value of miR-497-5p in HCC, we use bioinformatics and qRT-PCR assays to investigate the expression level of miR-497-5p in HCC tissues, as well as its association with overall survival and progress-free survival.

In our present study, the raw datasets from the GEO database and TCGA database were used to analyze miR-497-5p expression in HCC patients. The results demonstrated that the miR-497-5p expression level was significantly downregulated in HCC compared with adjacent normal tissues. A further correlation study showed that HCC with low miR-497-5p expression was significantly correlated with shorter OS and poor prognosis. In addition, we used quantitative real-time PCR to detect the expression of hsa-miR-497-5p in HCC biopsies and paracancerous tissues. This finding is consistent with our previous analysis data; the expression level of miR-497-5p in HCC was significantly downregulated contrasted with adjacent noncancerous tissues. Moreover, low expression of hsa-miR-497-5p correlated with poor clinicopathologic features, such as shorter OS and PFS. Furthermore, the correlation between hsa-miR-497-5p expression and clinical characters shows hsa-miR-497-5p expression downregulation and positive association with tumor diameter and negative association with OS and PFS. Our conclusions are consistent with previous studies; hsa-miR-497-5p is considered a tumor suppressor, which can inhibit the proliferation and invasion of tumor cells in HCC and NSCLC [97–99]. These results elucidated that the role of hsa-miR-497-5p exerts tumor-suppressive effects on clinical HCC samples.

In summary, our present study clarified the clinical and prognostic significance of hsa-miR-497-5p in HCC. The expression of hsa-miR-497-5p not only plays a significant role in the development and progression of HCC but also may serve as a potential diagnostic biomarker for HCC patients. The results also implied that hsa-miR-497-5p may be a promising and specific therapeutic target inductor of overexpression; cellular levels of miR-497-5p may be a new clinical therapeutic strategy for the treatment of HCC.

## Data Availability

All data generated or analyzed during this study are included in this article.
